# Weakly-Supervised Network for Detection of COVID-19 in Chest CT Scans

**DOI:** 10.1109/ACCESS.2020.3018498

**Published:** 2020-08-21

**Authors:** Ahmed Mohammed, Congcong Wang, Meng Zhao, Mohib Ullah, Rabia Naseem, Hao Wang, Marius Pedersen, Faouzi Alaya Cheikh

**Affiliations:** Department of Computer ScienceNorwegian University of Science and Technology (NTNU) 2815 Gjøvik Norway

**Keywords:** Attention, COVID-19, computed tomography, detection, enhancement, LSTM, weakly-supervised learning, deep learning, segmentation

## Abstract

Deep Learning-based chest Computed Tomography (CT) analysis has been proven to be effective and efficient for COVID-19 diagnosis. Existing deep learning approaches heavily rely on large labeled data sets, which are difficult to acquire in this pandemic situation. Therefore, weakly-supervised approaches are in demand. In this paper, we propose an end-to-end weakly-supervised COVID-19 detection approach, ResNext+, that only requires volume level data labels and can provide slice level prediction. The proposed approach incorporates a lung segmentation mask as well as spatial and channel attention to extract spatial features. Besides, Long Short Term Memory (LSTM) is utilized to acquire the axial dependency of the slices. Moreover, a slice attention module is applied before the final fully connected layer to generate the slice level prediction without additional supervision. An ablation study is conducted to show the efficiency of the attention blocks and the segmentation mask block. Experimental results, obtained from publicly available datasets, show a precision of 81.9% and F1 score of 81.4%. The closest state-of-the-art gives 76.7% precision and 78.8% F1 score. The 5% improvement in precision and 3% in the F1 score demonstrate the effectiveness of the proposed method. It is worth noticing that, applying image enhancement approaches do not improve the performance of the proposed method, sometimes even harm the scores, although the enhanced images have better perceptual quality.

## I. Introduction

COVID-19 has proliferated to more than 213 countries and territories in the world. The total number of reported cases until the time of writing (August 18, 2020) surpassed 21.9 million. Besides claiming lives of more than 774299, the cases are surging every day from almost every territory of the world [1]. The exponential human-to-human spread of the virus instigated worldwide apprehension which consequently forced the nations to take extreme measures in quest of effective solutions. Among the current diagnosis solutions, the real-time Reverse Transcription Polymerase Chain Reaction (rRT-PCR) test is the golden standard for COVID-19 confirmation. The rRT-PCR test is mainly done on respiratory samples obtained from people who have shown clinical symptoms [2]. However, the available rRT-PCR solutions have very high false-positive rates, which leads the suspected patients to be tested multiple times for achieving convincing diagnosis [3]. To efficiently utilize the scarce rRT-PCR resources as well as better accuracy in COVID-19 diagnosis, doctors are also relying on additional medical imaging technologies.

In the CT, COVID-19 manifests as a consolidated ground-glass opacity patch, scattered patches, and the thickening of interlobular septa on lung CT [4]. The lung lesions expand in size and density with the progression of the disease [5]. COVID-19 infected area of lung appears more contrasted than its surroundings in the chest CT. Primarily, such visibility in the respiratory system makes the chest CT suitable for diagnosing suspected COVID-19 cases [6], [7]. A recent literature review, conducted in March 2020, shows that “chest radiographs are of little diagnostic value in early stages, whereas CT findings may be present even before symptom onset” [5]. However, the aggressively growing number of suspected cases and the limited availability of medical diagnostic methods and resources have been putting pressure on medical professionals all over the world. To significantly alleviate the diagnostic workload, computer vision researchers have recently proposed viable solutions for detecting COVID-19. Among the proposed solutions, Artificial Intelligence (AI)-based chest CT analysis is playing an important role in fighting the COVID-19 pandemic [8]. AI-based diagnosis [9] is a supplementary assistance tool for radiologists, who usually have to analyze a large number of CT scans for diagnosis on a daily basis.

The AI-based analysis of chest CT for probable COVID-19 prevalence involves multiple steps from image acquisition, image pre-processing, segmentation, and final diagnosis [10]. However, the recent deep learning-based approaches require labeled datasets to train models. Since the labeling process of CT scans requires expert knowledge (mainly from a radiologist) and a significant amount of time, most of the supervised learning-based models are trained on a limited amount of data. Fully supervised methods that are trained on insufficient data usually are limited in their performance [11]. Therefore, a weakly-supervised approach for COVID-19 detection from weakly labeled data is indispensable. Following a similar approach to [12]–[14], we propose a weakly-supervised approach to detect the pathology of COVID-19 in the individual slices of CT scan using only volume level data labels. More precisely, a Convolutional Neural Network (CNN) named ResNext+ is proposed that integrates a lung segmentation mask with the corresponding CT volume and extracts spatial features from the CT volume. Additionally, a spatial and channel attention module is Incorporated in Restnext+ architecture for refining the feature maps. Then bidirectional LSTM is exploited for the axial dependency of the input slices. Essentially, the bidirectional LSTM transforms the spatial features to spatial-axial features. After that, a slice attention module is introduced to weight the importance of each slice and finally, a fully connected layer is utilized for the final slice and volume level prediction. Furthermore, as a pre-processing, two enhancement approaches are exploited for improving the accuracy of the model. In a nutshell, the contributions of the proposed method are threefold:

• We designed an end-to-end framework that is capable of learning from weakly labeled data. The network consists of a convolutional neural network named ResNext+ that takes a CT slice together with a binary mask of the lung section as input and fuses both pieces of information and gives spatial features of the CT volume. Channel and spatial attention modules are incorporated in an end-to-end fashion that helps refine the feature maps. Additionally, a bidirectional LSTM is exploited for transforming the spatial features into spatial-axial features.
• For enhancing the quality of slices, two types of enhancement algorithms namely stochastic sampling and tone mapping are exploited that specifically highlight the details inside the lung region for assisting network training and diagnosis.
• We introduced slice attention that helps the network to focus on the semantic slices for the final inference. In addition to volume level prediction, the slice attention network gives the slice level prediction which helps in localizing the infected region of the lung due to COVID19.

## II. Related Work

Several imaging modalities including x-ray [15], [16], CT [6], [7], [17]–[21], and ultrasound [22], [23] have been employed for diagnosis of COVID-19. These imaging modalities can be used with the increasing number of deep learning-based COVID-19 detection methods. Usually, a pre-processing step like haze removal [24], enhancement [25], etc. are preceded by deep learning algorithms. In this work, the proposed approach is primarily intended for diagnoses of COVID-19 from chest CT images of suspected patients. The current state-of-the-art regarding deep learning-based diagnosis on CT scan images is mostly focused on the detection of lung nodules and COVID-19 diagnosis. Setio *et al.* [26] presented a nodule detection method where 2D patches of the candidate nodules in lung CT are extracted from multiple planes. The network contains various streams of 2D ConvNets for each patch from the lung volume. Later, their outputs are fused to obtain the final classification. Similarly, Xie *et al.* [27] introduced a nodule detection methodology improving the Fast Region-based Convolutional Network (R-CNN) network [28] through the introduction of two region proposal networks. The proposed network concatenates relevant information from the lower layer and their deconvolution layer to yield candidate nodules [29]. They used VGG16 [30] for feature extraction. Additionally, they incorporated the 3D input data contextual information that is generated by systematically training three separate models on three types of slices and finally fused the results. It is also mentioned that their model is trained again with the wrongly classified samples for improving the accuracy of the algorithm. To reduce the rate of fast positive, a novel architecture named ZNET [31] is introduced that uses two CNNs; one for obtaining candidate nodules and the other for reducing false positives [32]. The authors used UNet to generate a probability map based on which candidate nodules are acquired in axial slices. Subsequently, they generate candidate masks through thresholding. The LUNA16 challenge evaluation finally indicated that ZNET outperformed several other methods [32].

The success of such approaches leads to the successful deployment of several AI-based commercial CT platforms in combating COVID-19 [15], [33]. A comprehensive review of AI techniques in image data acquisition, segmentation, and diagnosis of COVID-19 is presented in [8]. The state-of-the-art AI-assisted diagnosis approaches can be partly grouped into three categories. A brief overview of each category is given in the following.

### A. Classification of COVID-19 Versus Non-COVID-19

Some of the recent studies aim to discriminate COVID-19 patients from non-COVID-19 ones. Most of these methods are based on different variant of UNet [34]. For example, the algorithm proposed by Chen *et al.* [35] and Zheng *et al.* [36], [37] mainly utilize U-Net, U-Net++ [38], and U-Net+3D based model architectures. The architecture of UNet has been used in different networks for the region of interest extraction, predicting suspicious lung regions, segmentation, and other related tasks. Among the different tested segmentation models such as U-Net [34], V-Net [39], FCN-8s [40], and 3D U-Net++ [38], 3D U-Net++ is reported to yield the best performance for segmentation [37]. Also, the combination of 3D U-Net++ segmentation with ResNet-50 model [41] has shown to provide better classification compared to other models like DPN-92 [42], Inception-v3 [43] and Attention ResNet-50 [44]. Most CNN models proposed for lung segmentation and COVID-19 diagnosis are trained on slice [45] or volume levels [36], thus can predict slice or volume levels scores, respectively. In such approaches, after slice prediction blocks, the slice scores are mostly fused to come up with case-level diagnosis.

### B. Classification of COVID-19 Versus

OTHER VIRAL PNEUMONIA

On many occasions, the appearance of COVID-19 lung infections and those of other pneumonia cases are quite similar [46] in the CT image. Therefore, the discrimination between COVID-19 and other pneumonia cases would be of great importance in clinical practice [46], [47]. Thus, many researchers have recently looked into AI-based classification solutions. For example, Wang *et al.* [48] used transfer-learning by adapting the inception neural network for the classification of COVID-19 cases from the other viral pneumonia cases. They trained their model with a total of 217 pathogen-confirmed Region Of Interest (ROI) images that are extracted from 99 collected CT images (44 COVID-19 and 55 other viral pneumonia cases). With 236 ROI images as the testing set, accuracy of 73.1%, specificity of 67%, and sensitivity of 74% were achieved. Similarly, Ying *et al.* [49] developed a deep learning-based CT diagnosis and lesions localization system (named DeepPneumonia). Their proposed network contained a Details Relation Extraction neural Network (DRE-Net) to obtain image-level predictions and an aggregation step to get case-level labels. A total of 1990 CT images (obtained from 88 COVID-19 patients, 101 bacteria pneumonia patients, and 86 healthy cases) were used for training and testing. The network gave a result with an Area Under the Curve (AUC) of 0.92 in image-level and with an AUC of 0.95 and a recall of 0.96. Shi *et al.* [50] and Xu *et al.* [51] proposed screening models to distinguish COVID-19 out of community-acquired and Influenza-A viral pneumonia. Shi *et al.* [50] segmented CT images using VB-Net [52] (a modified version of V-net [39]) and extracted location-specific features from: volume, infected lesion number, histogram distribution, and surface area. Machine-learning methods were then applied to decide the best features and later predict COVID-19 patients from community-acquired pneumonia patients. Their results were based on 2685 CT images. Out of 2685, 1658 were confirmed COVID-19, and 1027 were pneumonia cases. They achieved a sensitivity of 0.907, specificity of 0.833, and an accuracy of 0.879 under five-fold cross-validation. Xu *et al.* [51] employed multi CNN models with location-attention mechanism. A total of 618 CT samples (219 COVID-19, 224 Influenza-A viral pneumonia, and 175 healthy cases) were used to achieve an average F1-score of 0.856 for all the three categories.

### C. Severity Assessment of COVID-19

Besides the identification of COVID-19 from other pneumonia cases, severity assessment has been a recent research focus. From a study on CT images of recovered COVID-19 patients, four stages of lung patterns were identified. The patterns, termed as early (0 to 4 days after the initial symptom), progressive (5 to 8 days), peak (9 to 13 days), and absorption stages (more than 14 days) [4] provide important evidence for the necessity of CT-based COVID-19 severity assessments. In this regard, Xiong *et al.* [53] analyzed 42 patients of COVID-19 with both the initial and follow-up CT images to assess the severity and the progression of COVID-19. Correlations were evaluated among clinical, laboratory findings, and CT features. Linear regression analysis was used to identify the significant indicating variables for the severity progression of COVID-19. Additionally, another recent COVID-19 severity assessment based on the random forest method is proposed by Tang *et al.* [54]. The author’s analysis and three-fold validation on their extracted 63 quantitative features of the chest CT images gave an accuracy of 0.875, a true positive rate of 0.933, and a true negative rate of 0.745.

Most of the diagnosis systems report pleasing results with high detection and classification accuracy. However, the majority of methods rely on fully supervised learning, both on volume level and slice level. Such supervised methods require time and resources of experts for data labeling. To address such issues, our proposed framework is based on a weakly-supervised attention based network that performs slice level inference with only volume level data labels.

## III. Methodology

**FIGURE 1.**
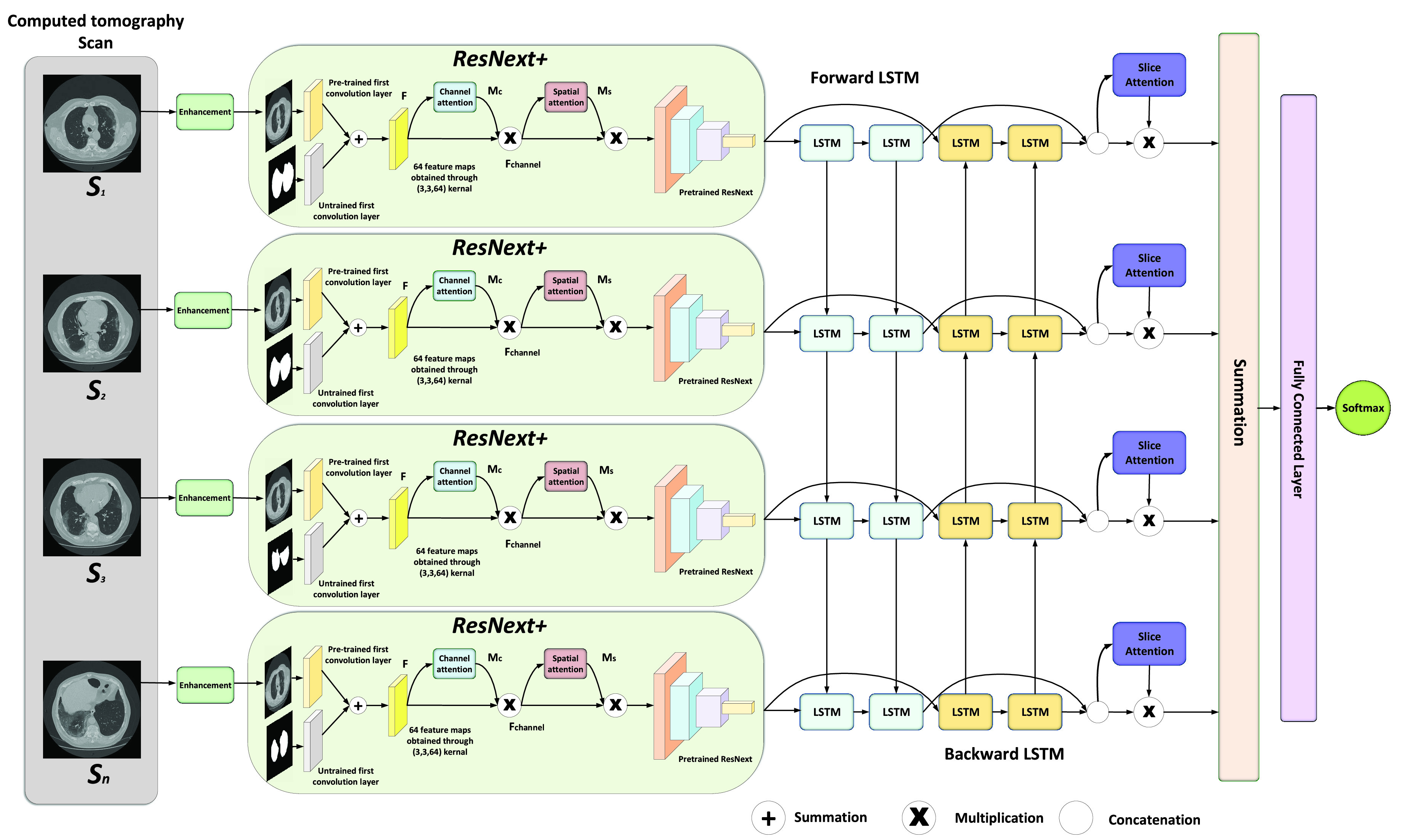
The CT slices are enhanced through the enhancement module and later spatial features are extracted through the ResNext+. The extracted spatial features are processed by bidirectional LSTM that transforms the spatial features to spatio-axial features and later refined by slice attention. The feature vector of each slice is summed and classified by a fully connected layer which is followed by a softmax that outputs underlying disease probabilities for the CT scan.

The proposed framework is shown in Fig. 1. The approach is motivated by a weakly-supervised capsule video endoscopy classification described in [12], [13] applied to CT volumes. The framework processes the whole CT volume and performs four discrete steps in an end-to-end fashion. Initially, the individual slices of the CT scan are fed to the proposed ResNext+ network for extracting spatial features. A brief description of the ResNext+ is given in Section III-B. Once the spatial features are extracted from the individual slice, the feature maps are given to the bidirectional LSTM. The bidirectional LSTM exploits the axial dependency in the input slices and transforms the spatial features to spatio-axial features. A detailed description of the attention module is given in Section III-C. Each LSTM (forward and backward) gives a spatio-axial feature vector of dimension $1\times 512$ which is concatenated and produces a spatio-axial feature vector of dimension $1\times 1024$. As the feature maps are spatially refined by the channel and spatial attention in the ResNext+, the resulted spatio-axial features are refined by the slice’s attention. Hence, in the third step of processing, the slice attention weights the importance of each slice for the inference. In the last step, a fully connected layer with 1024 hidden nodes and 2 output nodes classify the spatio-axial feature vector. To enhance the quality of the input slices, we adopted two enhancement techniques. A brief overview of the enhancement strategies is given in Section III-A.

### A. Enhancement

Accurate image-based disease diagnosis requires high-quality image data. CT images sometimes have low contrast that may hamper the visualization of critical structures. Moreover, it also affects the performance of deep learning algorithms as low contrast and suppressed details can make feature extraction difficult [55]. This motivates to apply image enhancement to the data before inputting it to the network. Enhancement of the medical images has a twofold impact on any automated disease detection framework. Firstly, enhancing the visual quality of input data improves visualization of significant pathologists; secondly, it improves the performance of feature extraction and segmentation algorithms [56]. Therefore, in this work, two image enhancement strategies (stochastic and tone mapping) were evaluated with the motivation to improve the feature learning of the proposed technique.

A brief description of the two enhancement approaches is given in the stochastic enhancement (Section III-A1) and tone mapping (Section III-A2) sections. Visual inspection (Fig. 7) of the enhancement results reveals that the methods lead to a well-contrasted lung area from the nearby bones and non-lung tissues. Moreover, the details of the infectious area are also improved as can be seen in the right lung (top row). Since the main focus of this work is not on visual quality, we have only investigated the impacts of the two enhancement methods on the performance of the proposed COVID-Attention-Net in terms of sensitivity, specificity, accuracy, precision, and recall. The detailed experimental analysis of COVID-Attention-Net is presented in Section V.

**FIGURE 2.**
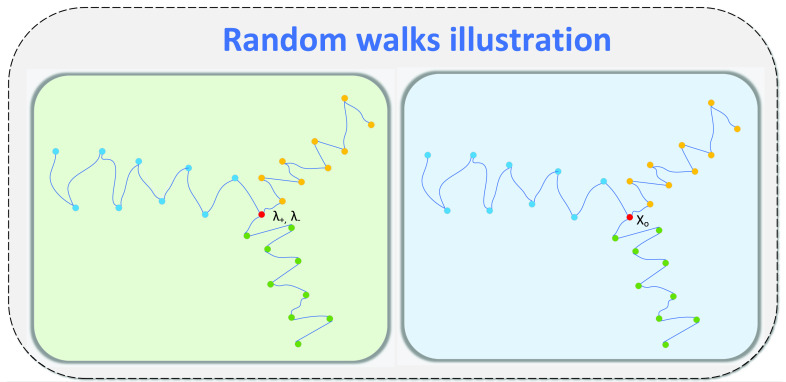
Illustration random walk: For each target pixel $x_{0}$, a random walk is initialized to compute intensity similarity and total variation of the gradient $(\lambda _{-}, \lambda _{+})$ along the random walk neighboring pixels $\mathbf {x_{j}}$. For clarity, in the figure, the number of iterations is $n=3$, while the number of samples is $M=9$.

**FIGURE 3.**
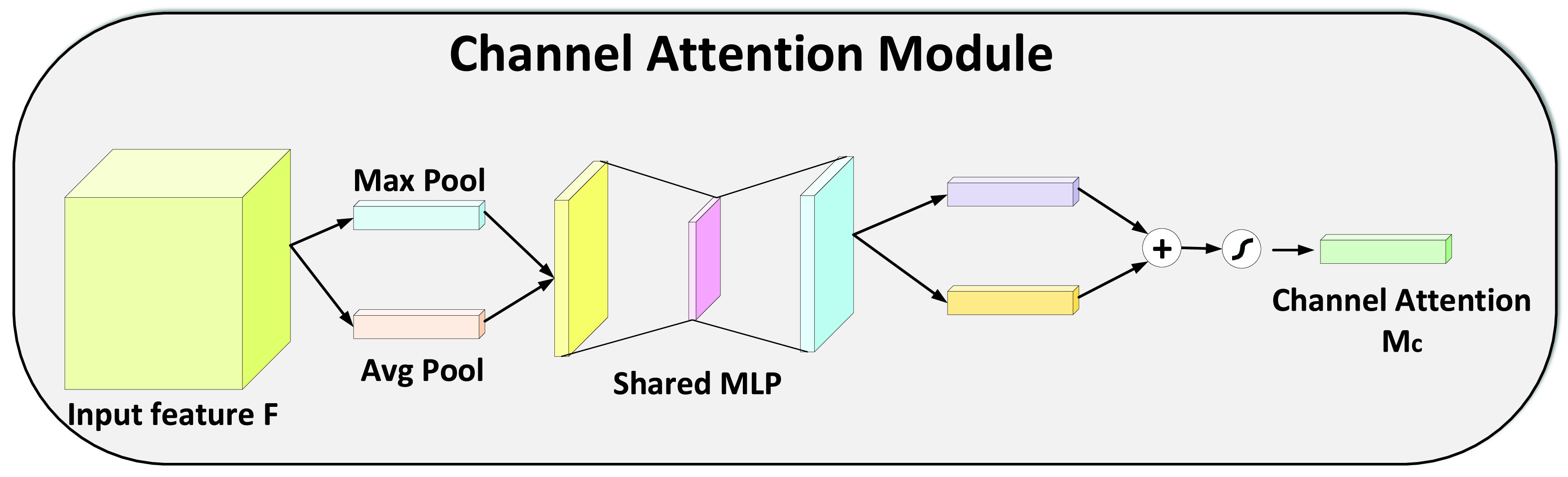
Illustration of Channel Attention Module.

**FIGURE 4.**
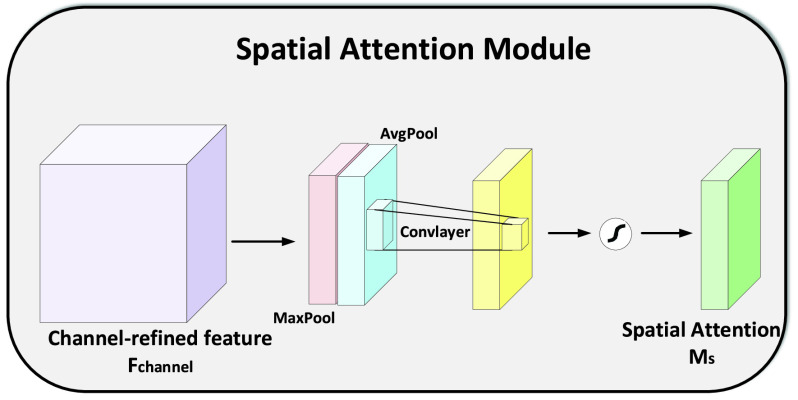
Illustration of Spatial Attention Module.

**FIGURE 5.**
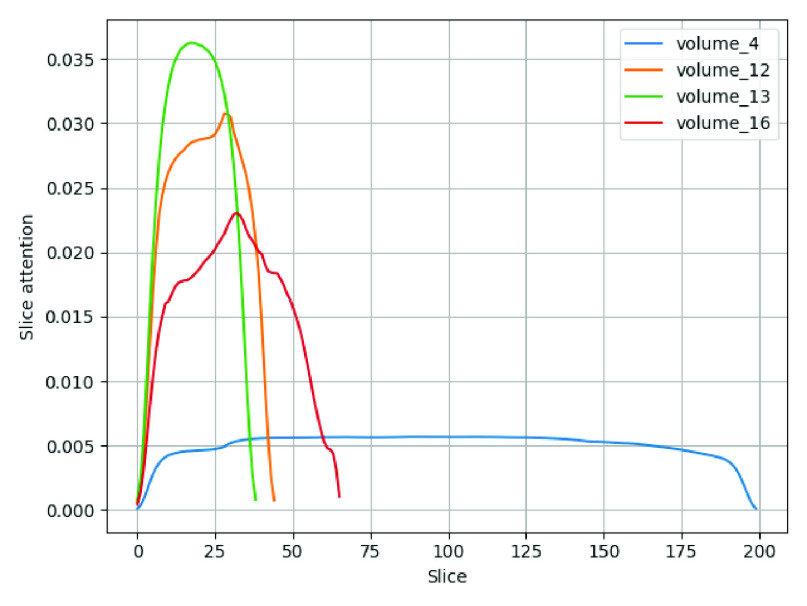
Response of slice attention on different CT volumes.

**FIGURE 6.**
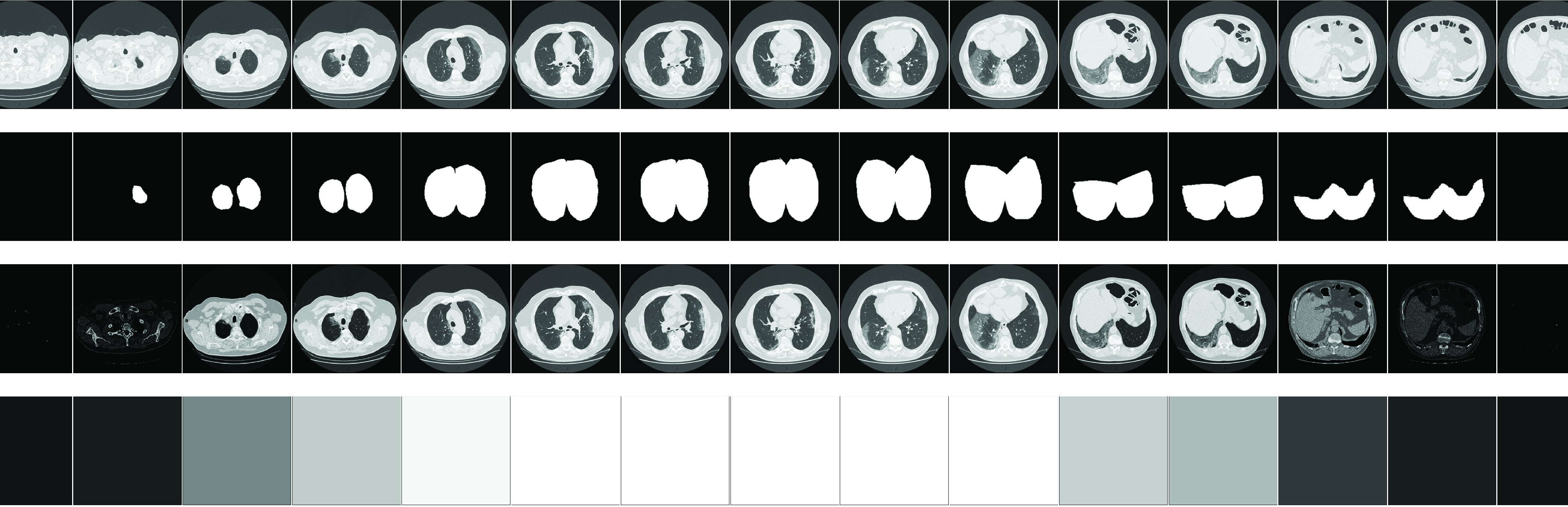
The top two rows show the original slices with their corresponding mask for Volume 12 in the test set. The third row shows the output of the slice attention combined with the original slice encoded as $\left({I^{ 0.001 +\frac {1}{\alpha _{n}}}}\right)$ for better visualization, where $\alpha _{n}$ is given by Eq. (19). The last row shows grayscale coded slice with black the irrelevant slices and white the relevant slices. Attention slices vary from Blank (Black) to the original frame corresponding to low and high value of attention weights, respectively. As it can be seen the network is able to localize slices containing COVID-19 slices (from the fourth column to the penultimate one).

**FIGURE 7.**
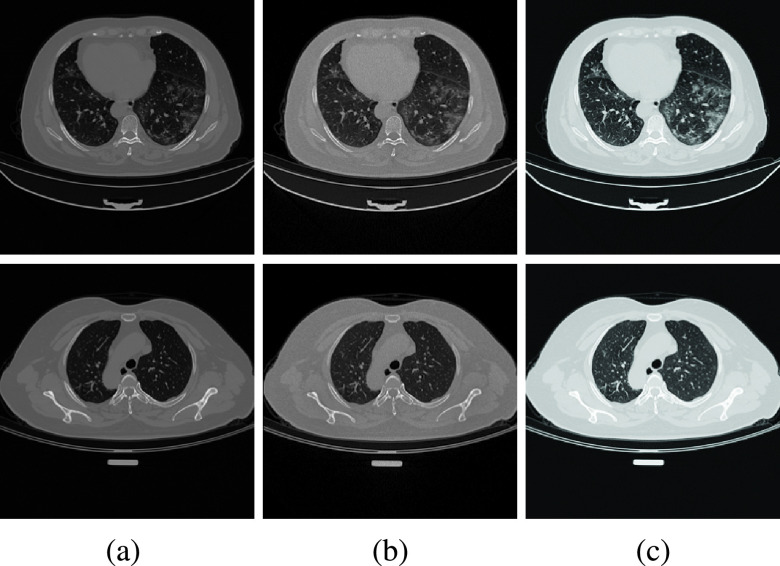
Qualitative results of the enhancement methods: (a) Original image. (b)Image enhanced by stochastic enhancement method [25]. (c) Image enhanced by tone mapping.

#### 1) Stochastic Enhancement

One of the evaluated enhancement methods is the stochastic sampling-based image enhancement algorithm proposed by Mohammed *et al.* [25] that helps in highlighting the lung tissues and the bronchioles in the CT images. The approach explores the local neighborhood of a pixel in two capacities. The algorithm analyzes the intensity similarity between the target pixel and the neighboring pixels which are characterized by the gradient between target pixel, the neighboring pixel, and their intensity difference. First, the image is decomposed into two layers $D_{1}$ and $D_{2}$. The local lightness and darkness contrast image $D_{1}$ is approximated with stochastic sampling and image local details $D_{2}$ are computed locally through the random walk. The enhanced CT image is given by:

\begin{align*} I_{enh}=&KD + I_{base} \\ D=&\gamma {D_{1}} + (1 - \gamma){D_{2}}\tag{1}\end{align*}

where $\gamma $ is a mixing coefficient that controls the amount of local details against image contrast, $K$ is a scalar constant and $I_{base}$ is the base layer.

To compute the base layer of the image $I_{base}$, for each pixel $x_{0}$ in the image, neighboring pixels are sampled with $M$ number of random walk. The random walk sampling is initialized at $x_{0}$ pass through random neighboring pixels $\mathbf {x_{j}}=\{x_{0}, x_{1}, {\dots } x_{n}\}$ on the $j_{th}$ random walk. The similarity of the target pixel $x_{0}$, to the neighboring pixels $\mathbf {x_{j}}| j \in M$ is expressed by a weighting function $\mathbf {w_{0}}^{j}(x_{0}|\mathbf {x_{j}})$ expressed as:

\begin{equation*} \mathbf {w_{0}}^{j}(x_{0}|\mathbf {x_{j}}) = \exp {\left ({- \frac {\left \|{x_{0}-\mathbf {x_{j}}}\right \|_{1}} {|\sigma _{I}| } - \frac {\left \|{TV(\nabla I)}\right \|_{1}}{ | \sigma _{g} | } }\right)}\tag{2}\end{equation*}

where $x_{0}$ is the target pixel and $\mathbf {x_{j}}$ corresponds to the set of intensity values of the neighboring pixels on $j_{th}$ random walk. Similarly, $\sigma _{I}$ and $\sigma _{g}$ are the normalization constant. Hence, the first term of the exponential represents the $l_{1}$ norm of the intensity difference between the initial pixel $x_{0}$ and neighboring pixel $\mathbf {x_{j}}$ normalized by the constant $\sigma _{I}$. The second term represents the total variation of eigenvalues of the structural tensors at each pixel normalized by the constant $\sigma _{g}$. The total variation term measures whether the random walk has crossed edges or not. This can be formulated using eigenvalues of the structural tensors $\lambda _{+}$ and $\lambda _{-}$ at each pixel. Using similar notation, the random walk gradient sampling is initialized with eigenvalues $(\lambda _{+}^{0},\lambda _{-}^{0})$ at the target pixel $x_{0}$ pass through random neighboring pixels $\mathbf {x_{j}}=\{(\lambda _{+}^{0},\lambda _{-}^{0}), (\lambda _{+}^{1},\lambda _{-}^{1}), {\dots }(\lambda _{+}^{n},\lambda _{-}^{n})\}$ on the $j_{th}$ random walk. Mathematically, the total variation term is defined as a sequence $TV(\nabla I) = \{TV(\nabla I)_{0}, TV(\nabla I)_{1}, {\dots } TV(\nabla I)_{n}\}$ where $TV(\nabla I)_{n}$ is given by:

\begin{equation*} TV(\nabla I)_{n} = \sum _{i=0}^{\mathbf {n}}\left ({\sqrt {(\lambda _{+}^{i+1}-\lambda _{-}^{i+1})} - \sqrt {(\lambda _{+}^{i}-\lambda _{-}^{i})} }\right)\tag{3}\end{equation*}

where $\lambda $’s are the eigenvalues of the structural tensors at each pixel which captures the dominant orientation of all neighboring pixel $\mathbf {x_{j}}$. Finally, each pixel in the base layer is computed as:

\begin{equation*} {x_{den}} = {\frac{{ {\sum \limits _{j = 0}^{M} {w_{0}^{j}({x_{0}}|{\mathbf {x_{j}}}){x_{0}}} } } }{ {{\sum \limits _{j = 0}^{M} {w_{0}^{j}({x_{0}}|{\mathbf {x_{j}}})} } }}}\tag{4}\end{equation*}

Eq. 1 and Eq. 4 summarize our enhancement. A graphical depiction of random walks is illustrated in Fig. 2. We applied this enhancement on all the slices of the CT scan as a pre-processing step for the deep network.

#### 2) Tone Mapping

The second approach we consider for CT image enhancement is through tone mapping operators. In some imaging conditions, the linear transformation of raw CT images (usually in 16-bit and high dynamic range format) to some of the common 8-bit (low dynamic range) image formats leads to loss of important image details. In different imaging applications such as high dynamic range image reproduction, several tone mapping, and contrast stretching operations need to be applied to compress the images’ dynamic range, while selectively preserving important image details [57]. Tone mapping operators have shown to be useful for CT images [58]. Therefore, we have tested a combination of global gamma and sigmoidal tone mapping operators for the preservation and enhancement of contrast around the lung regions of the CT scans, during image format conversion.

The CT images are stored as Digital Imaging and Communications in Medicine (DICOM) format 16-bit greyscale images with the pixel intensity proportional to tissue density represented in Hounsfield Unit (HU). A predefined threshold value of −600 HU is typically used to locate lung tissue [59]. Since most of the lung regions are represented by the lower mid of the intensity levels, we have applied inverse gamma followed by a sigmoid contrast enhancement function as given below:

\begin{equation*} I_{out} = \frac {1}{ 1 + e^{-a (I_{in})^{1/\gamma }}}\tag{5}\end{equation*}

In Eq. 5, the inverse gamma is $\gamma = 1.5$ and $a = 0.35$. As it can be seen from the resulting images, shown in Fig. 7, the two operations globally scaled the lightness value of the images in such a way that the darker regions (mainly lungs) of the images remain enhanced while suppressing the brighter regions (bones and other related organs) [60].

### B. ResNext

The architecture of ResNext+ is inspired by the classical ResNext [61]. However, it is different from classical ResNext in two ways. First, ResNext+ is capable of fusing the original slice with the corresponding binary mask, in our case of the lung region, in the first layer of the network. For the fusion, we used both the pre-trained and the untrained convolution layer of classical ResNext. The choice is motivated by the fact that ResNext is originally trained on the Imagenet dataset which consists of natural images. So, it is logical to use an untrained layer for the binary mask and a pre-trained layer for the slice. The second key attribute of the ResNext+ comes from the introduction of the channel and spatial attention. Channel and spatial attention have shown substantial improvement in several vision problems [62]. The key idea of the attention module is to refine the feature map and to give consciousness to the network regarding the important regions in the slices for the inference. A detailed description of the attention module is given in Section III-C.

### C. Attention Module

Designing a deep network with high performance and few parameters is one of the goals of the researchers in the community. Primarily, the most intuitive ideas like increasing the depth [63], [64], and width [43], [65] of the network is a well-adopted trend. However, the focus is shifting to the cardinality [61], [66] and the attention mechanism. Attention is mainly inspired by the human visual system. It is a relatively new term that is applied to deep models for improving the representation capability of the network and also helped the network to focus on the most important features. In our work, we exploited the Convolutional Block Attention Module (CBAM) [62] for fusing the cross-channel and spatial information in a given slice. Unlike [62], in our proposed method, cross-channel and spatial attention is applied only after the fusion of the slice and the binary mask. The CBAM improves the information flow from the layers of the network which consequently helps in information accentuation or suppression and as a result, gives a better representation for the infection prediction. For a given set of 64 feature maps $F\,\,\in \,\,\mathbb {R}^{C\times H\times W}$, the attention module extracts a 1D channel attention map $M_{c} \in \mathbb {R}^{C\times 1\times 1}$ and a 2D spatial attention map $M_{s} \in \mathbb {R}^{1\times H\times W}$ as shown in Fig. 3 and Fig. 4, respectively. Mathematically, it can be represented as:

\begin{align*} F_{channel}=&M_{c}(F) \otimes F \tag{6}\\ F_{spatial}=&M_{s}(F_{channel}) \otimes F_{channel}\tag{7}\end{align*}

where $F$ is the set of feature maps obtained after applying the first convolution and fusion (II-C), $F_{channel}$ is the channel attention feature maps and $F_{spatial}$ is the refined spatial attention feature maps. $\otimes $ indicates the element-wise multiplication.

#### 1) Channel Attention

The basic idea of channel attention is to find out what are the most important feature maps in the input volume. For the channel attention, we followed a similar formulation to that of [62] and used average pooling and max-pooling for squeezing the spatial dimension of the input feature maps. The averaged pooled $F^{c}_{avg}$ and max-pooled $F^{c}_{max}$ features are forwarded to a fully connected Multi-Layer Perceptron (MLP) with one hidden layer that generate the channel attention map $M_{c} \in \mathbb {R}^{C\times 1\times 1}$. The channel attention mechanism can be summarized as:

\begin{equation*} M_{c}(F) = \sigma (MLP(Avg_{pool}(F)) + MLP(Max_{pool}(F)))\tag{8}\end{equation*}

$F\,\,\in \,\,\mathbb {R}^{C\times H\times W}$ is the feature maps obtained through the CNN while $Avg_{pool}$ and $Max_{pool}$ are the average and max pooling operations, respectively.

\begin{equation*} M_{c}(F) = \sigma (W_{1}(W_{0}(F^{c}_{avg})) + W_{1}(W_{0}(F^{c}_{max})))\tag{9}\end{equation*}

The Sigmoid function $\sigma $ is used as the main activation function for the channel attention module. $W_{0} \in \mathbb {R}^{C/r \times C}$ and $W_{1} \in \mathbb {R}^{C \times {C/r}}$ are the input to hidden layer and hidden layer to output weight parameter for the MLP. For keeping the parameters of the MLP small, the hidden layer activation size is set to $\mathbb {R}^{C/r\times 1\times 1}$ with $r$ as the reduction ratio.

#### 2) Spatial Attention

Compared to channel attention, spatial attention aims to localize the most informative part of the feature maps that’s complementary to channel attention. To calculate the spatial attention, first average pooling and max pooling operations are applied to the feature maps and then the resulting feature maps are concatenated to get an efficient feature descriptor. On the resulting feature descriptor, a convolution layer is applied to generate the spatial attention map $M_{s}(F) \in \mathbb {R}^{H\times W}$. Mathematically, it can be defined as:

\begin{align*} M_{s}(F)=&\sigma (Conv^{9\times 9}([Avg_{pool}(F);Max_{pool}(F)])) \tag{10}\\ M_{s}(F)=&\sigma (Conv^{9\times 9}([F^{s}_{avg};F^{s}_{max}]))\tag{11}\end{align*}

where $F^{s}_{avg} \in \mathbb {R}^{1\times H\times W}$ and $F^{s}_{max} \in \mathbb {R}^{1\times H\times W}$ are the average and max pooling, respectively. The Sigmoid function $\sigma $ is used as the main activation function, while $Conv^{9\times 9}$ shows the convolution operation with a filter size of $9\times9$. The refined feature map is used as the slice descriptor and given to the bidirectional LSTM as the input.

## IV. Long-Short Term Memory

A feed-forward neural network [67] output solely based on the input data. In contrast, a recurrent neural network [68] has an internal memory where it stores the results of the previous samples. Hence, in the recurrent network, the output at any time instant $t$ not only depends on the input but also on the previous outputs of the network. Long-Short Term Memory (LSTM) is a special type of recurrent neural network that can retain past information for a longer period. The LSTM uses gates that can be seen as the information gateway that allows how much information can flow to the cell state through a sigmoid/hyperbolic tangent activation and a point-wise multiplication operation. The state of the LSTM cell is essentially the mechanism of storing the previous knowledge and a way of propagating only the useful knowledge to the next cells in the network. In an LSTM cell, the first two steps are related to calculating the information that needs to be kept in the cell state and the information that needs to be thrown away. Initially, the forget gate value is calculated as:

\begin{equation*} f_{t} = \sigma (W_{f}. [h_{t-1, x_{t}}] + b_{f}),\tag{12}\end{equation*}

where $W_{f}$ and $b_{f}$ are the learnable parameters and $x_{t}$ and $h_{t-1}$ are the input and the output of the previous state. Similarly, the input gate value is calculated as:

\begin{equation*} i_{t} = \sigma (W_{i}. [h_{t-1}, x_{t}] + b_{i}),\tag{13}\end{equation*}

with $W_{i}$ and $b_{i}$ the learnable parameter of the LSTM cell. Furthermore, an intermediate state value of the cell is also calculated from the current input $x_{t}$ and previous output $h_{t-1}$ and the learnable parameters $W_{c}$ and $b_{c}$ as:

\begin{equation*} \tilde {C_{t}} = \tanh (W_{c}. [h_{t-1}, x_{t}] + b_{c}),\tag{14}\end{equation*}

$\tilde {C_{t}}$ can be seen a raw state value that would be refined through the forget gate $f_{t}$ and the the input gate $i_{t}$ values at time $t$ as:

\begin{equation*} C_{t} = f_{t} * c_{t-1} + i_{t} * \tilde {C_{t}},\tag{15}\end{equation*}

Once the updated value of the state $C$ at time $t$ is calculated, the final output by the LSTM cell can be estimated in two steps. First an intermediate quantity $o_{t}$ as:

\begin{equation*} o_{t} = \sigma (W_{o} [h_{h-1}, x_{t}] + b_{o}),\tag{16}\end{equation*}

And based on $o_{t}$ and $C_{t}$, the final output is determined as:

\begin{equation*} h_{t} = o_{t} * \tanh (C_{t})\tag{17}\end{equation*}

The $h_{t}$ and $C_{t}$ act like the previous output and previous state of the next LSTM cell in the LSTM network. Bidirectional LSTMs are an extension to the LSTM network that enhances performance by feeding CT slices to two independent LSTM networks in forward and backward direction along the axial axis and concatenating the output features. Further analysis of LSTM is beyond the scope of the paper. For further details, the readers may refer to [69]–[72].

### 1) Slice Attention

Slice attention is the mechanism of enabling the network to focus mainly on the semantic slices to assist in modeling the axial dependencies in the data. The slice attention principle is similar to softmax function where the values are normalized and the sum is equal to 1. However, the slice attention of the individual slices shows the probability of slice having COVID-19. From the architectural point of view, slice attention is modeled as a two-layer fully connected neural network that takes the output of the bidirectional LSTM and gives the slice attention score. In our setting, the output of both the forward and backward LSTM is $1\times 512$ feature vectors which yield a feature vector of size $1\times 1024$ after concatenation. Mathematically, the slice attention is expressed as:

\begin{equation*} \mathcal {S} = \sum _{n=1}^{N} \alpha _{n} f_{n}\tag{18}\end{equation*}

where $\alpha _{n}$ is the attention response, $f_{n}$ is the input feature vector and $N$ is the number of slices considered for the inference. Given that the slice attention is a two-layer fully connected neural network, the attention response is obtained as:

\begin{equation*} \alpha _{n} = \frac {\exp (w^{T} \tanh (bf_{n}^{T}))}{\sum _{n=1}^{N} \exp (w^{T} \tanh (bf_{n}^{T}))}\tag{19}\end{equation*}

In Eq. 19, $w$ and $b$ are the parameters of the two-layer network and $N$ is the total number of slices. During the training, given the $\tanh $ activation function works for both the negative and positive values, the gradient of the cost function is back-propagated efficiently. Hence, it can be seen in Fig. 5 that slice attention is producing an effective response by computing an adaptive weighted average of the bidirectional LSTM features. The accumulated slice attention response $\mathcal {S}$ is passed through a fully connected layer with 1024 hidden nodes and two output nodes that yields $v_{i}$; the volume response, that is consequentially given to a 2-way softmax function for the final class probability inference.

## V. Experiments

A set of experiments has been performed to evaluate the performance of the proposed network without applying any kind of enhancement as well as after applying enhancement as described in the prior section. The experimental details, dataset, and underlying pre-processing steps are presented as follows.

### A. Dataset

To train and test the proposed COVID-Attention-Net, we used a total of 302 CT volumes (20 with confirmed COVID-19 patients) consisting of a total 3520 positive and 19,353 negative cases slices. The positive and negative CT data is acquired from Joseph^1^ *et al.* [73] (collected from a few Chinese, Iranian and Italian hospitals) and Tianchi Lung diseases diagnosis competition CT images,^2^,^3^ respectively. Two radiologists assisted in the manual annotation of 20 positive cases both at volume and slice level, though the slice level annotations are only used for performance evaluation purposes. The dataset is finally split into training and testing sets with the random 80/20 ratio and the difficulty level of both sets are confirmed to be balanced by the radiologist. The CT scans, originally existing in mhd or nifty formats, are linearly transformed to the standard grayscale intensity range. The positive and negative data were acquired from different sources and both had different dimensions, therefore all the 2D slices were resized to $256 \times 256$ to ensure the same spatial dimension for the whole input data.^1^https://academictorrents.com/details/136ffddd0959108becb2b3a86630bec049fcb0ff (Accessed: 10 July 2020)^2^https://tianchi.aliyun.com/competition/entrance/231724/introduction (Accessed: 10 July 2020)^3^https://aistudio.baidu.com/aistudio/datasetdetail/8689 (Accessed: 10 July 2020)

### B. Lung Mask Extraction

Most of the time, chest CT images contain lung and non-lung tissues such as bones and fat. Since COVID-19 effects can only be viewed in the lung region, we incorporated the lung mask in the first stages of the proposed network, as shown in Section III. Hence, the lung mask is extracted by binarizing the CT slices with a threshold of -600 HU, adapted from Liao *et al.* [74]. Then convex hull of the mask is computed, followed by a dilation operation to include the outer wall of the lung. The original CT slice and the corresponding estimated mask are shown in the first and second row of Fig. 6. For some challenging volumes, such as the lung part containing severe pathologies, the binarizing method may fail to segment the lung part. Therefore, those severe and wrongly segmented cases are manually checked and removed from the training dataset.

### C. Class Balancing and Data Augmentation

As described earlier, the numbers of positive (20) and negative (282) CT volumes are not balanced. Such type of long-tailed data distribution is a frequently encountered issue in several classification problems [75]. It adversely affects the sensitivity of the detection algorithm and consequently, the network either wrongly identifies majority true positives as false positive or true negative as a false negative. Class balancing strategies including resampling, oversampling, and cost-sensitive reweighting have been incorporated in several classification methods to resolve the class imbalance challenge and considerably improve the performance of an algorithm [76]. Resampling operates on data level and modifies the class distribution of training data. Considering the extreme imbalance between the quantity of negative and positive instances, we incorporate resampling strategy in case of positive instances. To further minimize the skewness in data distribution, we later apply data augmentation by introducing invariance in the positive samples. Intensity transformations including contrast stretching, the addition of Gaussian noise, blur, and spatial transformations such as zooming, scaling, rotation, and elastic deformation is applied to augment positive samples count. In this way, we increase the intra and interclass disparity in our dataset.

### D. Implementation Details

Our method is implemented with PyTorch library [77] and trained on a single NVIDIA TITAN RTX GPU with 24GB graphic memory. For the stochastic image enhancement, the number of iterations $n$ are selected to be 20 while the number of samples $M$ in each iteration is fixed to 250. The normalization constant $\sigma _{I}$ and $\sigma _{g}$ are chosen empirically and fixed at 0.5 and 0.3, respectively. For all our experimental scenarios, we used pre-trained ResNext [61] convolutional layers to extract features from slices. The slices and their corresponding masks are resized to $224 \times 224$. We applied a $7 \times 7$ convolution to the slices and their corresponding masks before summation as shown in Fig. 1. The network is trained end-to-end with binary cross-entropy Eq. 20 and batch-size of 1, as each CT volume contains a variable number of slices. The Adam optimizer [78] with cyclic learning rate scheduling technique of $lr_{min} = 1e^{-5}$ and $lr_{max} = 1e^{-4}$ values is used for all our training [79]. The LSTM blocks are initialized from a normal distribution with 0 mean and 0.01 variance. We have also disabled all batch normalization layers running estimates and trained each experimental case for a total of 40 epochs.

\begin{equation*} l(g,p) = -g\log (p)-(1-g)\log (1-p)\tag{20}\end{equation*}

where $p = \frac {\exp {({-v_{i}})}}{\sum _{i=0}^{1} \exp {({-v_{i}})}} $ and $v_{i}$ is the output of the last fully connected layer and $g$ is the ground truth label.

Since the number of slices in each volume of our dataset ranges from 30 to 350 and due to the limited availability of single GPU memory, the number of input slices is restricted to a maximum of 50. Therefore, random sampling is done in our implementation to input 50 slices from a particular volume to feed the network.

### E. Experimental Results

To evaluate the performance of the proposed detection method, six commonly used evaluation metrics (Accuracy (ACC), Precision (PRE), F1-score (F1), Sensitivity (SEN), and Specificity (SPE)) computed from the confusion matrix between the ground-truth labels and the predicted labels are used. The performance is evaluated both on the volume and slice level. In order to analyze the role of each block of the proposed method, the following comparisons are done. First, the method is evaluated by modifying the enhancement block. In this regard, three experiments are implemented to analyze the performance of the network in terms of assessment metrics mentioned above by inputting data without applying any enhancement, after applying stochastic enhancement [25] and after applying tone mapping. Then, an ablation study of the network is conducted to evaluate the improvement in the performance of the network. The detail of the experiments and results are discussed and analyzed in the following section.

### F. Analysis of Image Enhancement

Stochastic enhancement and tone mapping, described in Section III-A, are applied to the original images to highlight details inside the lung area and therefore provide the network with more information. As shown in Fig. 7, after enhancement, the area of interest in the image can be seen more clearly without the introduction of any additional artifacts, especially in the COVID-19 infected lung area (first row).

Table 1 shows the detection results on the original and enhanced CT images, both on volume level and slice level. On volume level evaluation, images with or without enhancement all achieve a 100% performance in all the assessment metrics, which means that all positive cases are correctly detected. However, on the slice level, the sensitivity after stochastic enhancement improves by 2.8%, while the values of other evaluation metrics all dropped compared with the original images. There could be two possibilities; our model parameters are fine-tuned only on the original images instead of fine-tuning it separately for the original and enhanced images as input. The second could be that the enhancement is applied on whole slices instead of the lung area (the region of interest (ROI) in our case), which may enhance the non-lung areas, leading the incorrect image regions to receive more attention.

**TABLE 1:** Detection Results of Different Enhancement Methods

		ACC	PRE	REC	F1	SEN	SPE
Volume Level Evaluation	Orig.	1.0	1.0	1.0	1.0	1.0	1.0
	Stoc. Enh. [25]	1.0	1.0	1.0	1.0	1.0	1.0
	Tone Enh.	1.0	1.0	1.0	1.0	1.0	1.0
Slice Level Evaluation	Orig.	0.776	0.819	0.854	0.814	0.855	0.793
	Stoc. Enh. [25]	0.762	0.792	0.882	0.809	0.883	0.729
	Tone Enh.	0.729	0.788	0.773	0.761	0.773	0.772

### G. Ablation Study of the Network

To illustrate the effectiveness of each module included in the network, an ablation study is performed by removing some modules from the network while retaining the rest. We experimented with three configurations in order to analyse the impact of different components on the performance of the proposed network:

a). excluding spatial and channel attention (N-SCA)
b). excluding slice attention (N-SLA)
c). excluding the segmentation mask (N-MSCA).

The spatial and channel attention modules of the network are first removed from the original model and the results indicate that the network can precisely predict volume level COVID-19 with the proposed model and without spatial and channel attention (N-SCA). The spatial and channel attention modules can boost the slice level performance of the network by 2.17%, 5.2%, 2.6%, and 6.0% on ACC, PRE, F1, and SPE respectively. The scores of SEN also show a similar trend. This can be observed by comparing the results of proposed and N-SCA configurations in Table 2.

**TABLE 2:** Results of Ablation Study of the Proposed Approach. N-SCA: Without Spatial and Channel Attention; N-SLA: Without Slice Attention; N-MSCA: Without Segmentation Mask, Spatial and Channel Attention

		ACC	PRE	F1	SEN	SPE
Volume Level Evalution	N-SCA	1.0	1.0	1.0	1.0	1.0
	N-MSCA	0.933	0.00	0.00	0.00	1.0
	N-SLA	1.0	1.0	1.0	1.0	1.0
	Proposed	1.0	1.0	1.0	1.0	1.0
Slice Level Evaluation	N-SCA	0.755	0.767	0.788	0.856	0.733
	N-MSCA	0.788	0.763	0.834	0.975	0.626
	N-SLA	NA	NA	NA	NA	NA
	Proposed	0.776	0.819	0.814	0.855	0.793

When the lung segmentation mask is further removed from the proposed network in case of N-MSCA configuration (i.e. without segmentation mask, spatial and channel attention), the network performance drops to 93.3% for ACC, 0 for PRE, F1 and, SEN which indicates that the network classifies all the positive volumes as negatives. In other words, N-MSCA cannot detect the COVID-19 in the overall volume. It is worth pointing out that the N-MSCA obtains a comparably better result for the slice level by locating the important slices. However, even the ability of the network to focus better on the slice level does not contribute to the correct final prediction on the volume level. This experiment demonstrates the importance of the segmentation mask. Moreover, it emphasizes the significance of the spatial and channel attention modules in the proposed architecture as well.

In order to investigate the influence of the slice attention module on volume level, we conduct the third experiment (N-SLA configuration), where the slice attention of the network is removed from the proposed setup. The network obtained the same performance for volume level evaluation. However, without the slice attention module, it is not possible to localize the slices that contain COVID-19, which reduces the explainability of such approaches.

## VI. Discussion

In this work, we proposed a deep learning-based end-to-end framework that not only gives a volume level detection, but is also capable of classifying slices containing COVID-19 infection. Furthermore, we present the first study on COVID-19 detection employing weakly-supervised network using volume level labels to achieve slice level prediction.

### A. Effectiveness and Application

We demonstrate the effectiveness of our method on volume level and slice level prediction. Considering volume level diagnosis, we attain 100% performance on all evaluation metrics (with or without enhancement), implying that the proposed method can correctly detect all the COVID-19 cases on our test data. For slice level attention, the labeled data is used just for validation, which means that the model is unsupervised for making slice level prediction. Nevertheless, promising results are obtained with the proposed model, indicating that the attention modules can help to locate the more suspicious slices.

Our main goal is to assist the doctors in the diagnosis of COVID-19, so the application can be beneficial from two perspectives: First, at the volume level, the proposed network can give a pre-diagnosis for the doctors to identify the individual/overall suspected COVID-19 cases. Second, at the slice level, the slice attention can allow the doctors to only focus on the sensitive slices that are candidates of containing the COVID-19 infection instead of examining the entire volume.

### B. Limitations and Future Work

While the proposed approach shows an encouraging performance, there are several limitations regarding our dataset and methodology.

• Dataset limitations: The dataset in our study does not include common and other viral pneumonia, which is also important for COVID-19 detection. There are fewer COVID-19 positive cases compared to the negative cases which lead the dataset to class imbalance issues. We conducted re-sampling on the slice level to weaken the imbalance, but still, this problem introduces challenges on training and evaluation of the network.
• Methodology limitations: COVID-19 detection is a new emerging research field. Therefore, there is no standard dataset publicly available. Thus, the comparison of the proposed technique with state-of-the-art is not currently feasible. The evaluated stochastic enhancement is also applied to the gray-scale images, which may introduce information loss in the quantization step. Additionally, our network parameters are only fine-tuned on original images, which may not fully demonstrate the gain in network performance realized by including enhancement methods.

Considering such limitations of this study; we plan to improve our model in two ways in the future.

• Data acquisition and labeling: More COVID-19 and other pneumonia cases will be labeled and added to the dataset to demonstrate the robustness of our model and improve the data imbalance issue. Moreover, having a larger dataset will improve the attention. For example, narrower slice attention will be achieved with a larger dataset compared to the Gaussian like slice attention that is achieved from the current dataset. Narrower slice attention will help the doctor to pinpoint the slices with COVID-19 infection.
• Methodology improvement: Further investigation will be done regarding the application of enhancement on original images instead of re-scaled ones. A Comparison with the state-of-the-art will also be conducted once a standard public dataset is available.
• In the current study, the masks are annotated manually for training the network. However, with the availability of standard public datasets, the segmentation task can be learned and trained in an end-to-end fashion.

## VII. Conclusion

A weakly-supervised deep learning-based framework for COVID-19 detection is proposed in this paper. The proposed framework use combination of lung segmentation mask, attention aware mechanism, and LSTM for extracting the spatial, axial, and temporal features from the CT volume. Initially, resampling accompanied by data augmentation techniques is applied to address the scarcity and imbalance of binary class data distribution. As a pre-processing step, stochastic and tone-mapping based image enhancement methods were evaluated for performance improvement of the model. Finally, the performance evaluation of the proposed framework is conducted using several module configurations. The ablation study shows that the combination of all the attention modules and the segmentation mask yields the best performance. On volume level prediction, the proposed method achieved a 100% performance on all evaluation metrics and experimental cases. For slice level prediction, however, a different performance was observed in different experimental cases. In general, the integration of slice attention enables radiologists to focuse only on the salient areas of the whole CT volume. From clinical perspectives, the proposed framework can facilitate the prognosis of COVID-19 by radiologists. Moreover, it paves the way for future research targeted at COVID-19 detection from limited and weakly labeled data.

## References

[1] Johns Hopkins University. (2020). Coronavirus COVID-19 Global Cases by the Center for Systems Science and Engineering (CSSE). Accessed: Apr. 2, 2020. [Online]. Available: https://coronavirus.jhu.edu/map.html

[2] Centers for Disease Control and Prevention. (2020). Testing for COVID-19. Accessed: Apr. 2, 2020. [Online]. Available: https://www.cdc.gov/coronavirus/2019-ncov/symptoms-testing/testing.html

[3] T. Liang, “Handbook of COVID-19 prevention and treatment,” Zhejiang Univ. School Med., Hangzhou, China, Tech. Rep., 2020.

[4] F. Pan, T. Ye, P. Sun, S. Gui, B. Liang, L. Li, D. Zheng, J. Wang, R. L. Hesketh, L. Yang, and C. Zheng, “Time course of lung changes on chest CT during recovery from 2019 novel coronavirus (COVID-19) pneumonia,” Radiology, vol. 295, Feb. 2020, Art. no. 200370. 10.1148/radiol.2020200370 PMC7233367 32053470

[5] S. Salehi, A. Abedi, S. Balakrishnan, and A. Gholamrezanezhad, “Coronavirus disease 2019 (COVID-19): A systematic review of imaging findings in 919 patients,” Amer. J. Roentgenology, vol. 215, pp. 1–7, Mar. 2020. 10.2214/AJR.20.23034 32174129

[6] Y.-H. Jin, L. Cai, Z. S. Cheng, H. Cheng, T. Deng, Y. P. Fan, C. Fang, D. Huang, L. Q. Huang, Q. Huang, and Y. Han, “A rapid advice guideline for the diagnosis and treatment of 2019 novel Coronavirus (2019-nCoV) infected pneumonia (standard version),” Mil. Med. Res., vol. 7, no. 1, p. 4, 2020. 32029004 10.1186/s40779-020-0233-6 PMC7003341

[7] Y. Fang, H. Zhang, J. Xie, M. Lin, L. Ying, P. Pang, and W. Ji, “Sensitivity of chest CT for COVID-19: Comparison to RT-PCR,” Radiology, vol. 296, no. 2, Feb. 2020, Art. no. 200432. 10.1148/radiol.2020200432 PMC7233365 32073353

[8] F. Shi, J. Wang, J. Shi, Z. Wu, Q. Wang, Z. Tang, K. He, Y. Shi, and D. Shen, “Review of artificial intelligence techniques in imaging data acquisition, segmentation and diagnosis for COVID-19,” 2020, arXiv:2004.02731. [Online]. Available: http://arxiv.org/abs/2004.02731 10.1109/RBME.2020.2987975 32305937

[9] R. Lin, Z. Ye, H. Wang, and B. Wu, “Chronic diseases and health monitoring big data: A survey,” IEEE Rev. Biomed. Eng., vol. 11, pp. 275–288, Apr. 2018. 29993699 10.1109/RBME.2018.2829704

[10] J. Bullock, A. Luccioni, K. Hoffmann Pham, C. Sin Nga Lam, and M. Luengo-Oroz, “Mapping the landscape of artificial intelligence applications against COVID-19,” 2020, arXiv:2003.11336. [Online]. Available: http://arxiv.org/abs/2003.11336

[11] V. Cheplygina, M. de Bruijne, and J. P. W. Pluim, “Not-so-supervised: A survey of semi-supervised, multi-instance, and transfer learning in medical image analysis,” Med. Image Anal., vol. 54, pp. 280–296, 5 2019. 30959445 10.1016/j.media.2019.03.009

[12] A. Mohammed, I. Farup, M. Pedersen, S. Yildirim, and Ø. Hovde, “PS-DeVCEM: Pathology-sensitive deep learning model for video capsule endoscopy based on weakly labeled data,” Comput. Vis. Image Understand., 2020, Art. no. 103062.

[13] A. K. Mohammed, “Computational techniques for pathology detection and quality enhancement with emphasis on colonic capsule endoscopy,” Ph.D. dissertation, Dept. Comput. Sci., Norwegian Univ. Sci. Technol., Gjøvik, Norway, 2019.

[14] A. Kedir, M. Ullah, and J. R. Bauer, “Spectranet: A deep model for skin oxygenation measurement from multi-spectral data,” in Proc. Electron. Imaging Conf. Society for Imaging Science and Technology, 2020. [Online]. Available: https://www.ingentaconnect.com/content/ist/ei/pre-prints/content-ei2020-color-083

[15] United Imaging. (2020). United Imaging Sends Out More Than 100 CT Scanners and X-Ray Machines to Aid Diagnosis of the Coronavirus. Accessed: Apr. 8, 2020. [Online]. Available: https://www.itnonline.com/content

[16] I. D. Apostolopoulos and T. A. Mpesiana, “Covid-19: Automatic detection from X-ray images utilizing transfer learning with convolutional neural networks,” Phys. Eng. Sci. Med., vol. 43, no. 2, pp. 635–640, Jun. 2020. 32524445 10.1007/s13246-020-00865-4 PMC7118364

[17] D.-P. Fan, T. Zhou, G.-P. Ji, Y. Zhou, G. Chen, H. Fu, J. Shen, and L. Shao, “Inf-net: Automatic COVID-19 lung infection segmentation from CT images,” 2020, arXiv:2004.14133. [Online]. Available: http://arxiv.org/abs/2004.14133 10.1109/TMI.2020.2996645 32730213

[18] X. Ouyang, J. Huo, L. Xia, F. Shan, J. Liu, Z. Mo, F. Yan, Z. Ding, Q. Yang, B. Song, F. Shi, H. Yuan, Y. Wei, X. Cao, Y. Gao, D. Wu, Q. Wang, and D. Shen, “Dual-sampling attention network for diagnosis of COVID-19 from community acquired pneumonia,” 2020, arXiv:2005.02690. [Online]. Available: http://arxiv.org/abs/2005.02690 10.1109/TMI.2020.2995508 32730212

[19] J. Wang, Y. Bao, Y. Wen, H. Lu, H. Luo, Y. Xiang, X. Li, C. Liu, and D. Qian, “Prior-attention residual learning for more discriminative COVID-19 screening in CT images,” IEEE Trans. Med. Imag., vol. 39, no. 8, pp. 2572–2583, Aug. 2020. 10.1109/TMI.2020.2994908 32730210

[20] K. He, W. Zhao, X. Xie, W. Ji, M. Liu, Z. Tang, F. Shi, Y. Gao, J. Liu, J. Zhang, and D. Shen, “Synergistic learning of lung lobe segmentation and hierarchical multi-instance classification for automated severity assessment of COVID-19 in CT images,” 2020, arXiv:2005.03832. [Online]. Available: http://arxiv.org/abs/2005.03832 10.1016/j.patcog.2021.107828 PMC7816595 33495661

[21] S. Hu, Y. Gao, Z. Niu, Y. Jiang, L. Li, X. Xiao, M. Wang, E. Fei Fang, W. Menpes-Smith, J. Xia, H. Ye, and G. Yang, “Weakly supervised deep learning for COVID-19 infection detection and classification from CT images,” 2020, arXiv:2004.06689. [Online]. Available: http://arxiv.org/abs/2004.06689

[22] D. Buonsenso, A. Piano, F. Raffaelli, N. Bonadia, K. D. G. Donati, and F. Franceschi, “Novel Coronavirus disease-19 pnemoniae: A case report and potential applications during COVID-19 outbreak,” Eur. Rev. Med. Pharmacological Sci., vol. 24, pp. 2776–2780, 2020. 10.26355/eurrev_202003_20549 32196627

[23] S. Roy, “Deep learning for classification and localization of COVID-19 markers in point-of-care lung ultrasound,” IEEE Trans. Med. Imag., vol. 39, no. 8, pp. 2676–2687, Aug. 2020. 10.1109/TMI.2020.2994459 32406829

[24] S. Kuanar, K. R. Rao, D. Mahapatra, and M. Bilas, “Night time haze and glow removal using deep dilated convolutional network,” 2019, arXiv:1902.00855. [Online]. Available: http://arxiv.org/abs/1902.00855

[25] A. Mohammed, I. Farup, M. Pedersen, Ø. Hovde, and S. Y. Yayilgan, “Stochastic capsule endoscopy image enhancement,” J. Imag., vol. 4, no. 6, p. 75, Jun. 2018.

[26] A. A. A. Setio, F. Ciompi, G. Litjens, P. Gerke, C. Jacobs, S. J. van Riel, M. M. W. Wille, M. Naqibullah, C. I. Sanchez, and B. van Ginneken, “Pulmonary nodule detection in CT images: False positive reduction using multi-view convolutional networks,” IEEE Trans. Med. Imag., vol. 35, no. 5, pp. 1160–1169, 5 2016. 10.1109/TMI.2016.2536809 26955024

[27] H. Xie, D. Yang, N. Sun, Z. Chen, and Y. Zhang, “Automated pulmonary nodule detection in CT images using deep convolutional neural networks,” Pattern Recognit., vol. 85, pp. 109–119, Jan. 2019.

[28] R. Girshick, “Fast R-CNN,” in Proc. IEEE Int. Conf. Comput. Vis. (ICCV), Dec. 2015, pp. 1440–1448.

[29] S. E. Gerard, T. J. Patton, G. E. Christensen, J. E. Bayouth, and J. M. Reinhardt, “FissureNet: A deep learning approach for pulmonary fissure detection in CT images,” IEEE Trans. Med. Imag., vol. 38, no. 1, pp. 156–166, Jan. 2019. 10.1109/TMI.2018.2858202 PMC6318012 30106711

[30] K. Simonyan and A. Zisserman, “Very deep convolutional networks for large-scale image recognition,” 2014, arXiv:1409.1556. [Online]. Available: http://arxiv.org/abs/1409.1556

[31] Y. Zhang, J. Wu, W. Chen, Y. Chen, and X. Tang, “Prostate segmentation using Z-Net,” in Proc. IEEE 16th Int. Symp. Biomed. Imag. (ISBI), Apr. 2019, pp. 11–14.

[32] A. A. A. Setio, “Validation, comparison, and combination of algorithms for automatic detection of pulmonary nodules in computed tomography images: The LUNA16 challenge,” Med. Image Anal., vol. 42, pp. 1–13, 2017. 28732268 10.1016/j.media.2017.06.015

[33] Alibaba Cloud. (2020). CT image analytics for COVID-19. Accessed: Apr. 8, 2020. [Online]. Available: https://www.alibabacloud.com/zh/solutions/ct-image-analytics

[34] O. Ronneberger, P. Fischer, and T. Brox, “U-Net: Convolutional networks for biomedical image segmentation,” in Proc. Int. Conf. Med. Image Comput. Comput.-Assist. Intervent. Cham, Switzerland: Springer, 2015, pp. 234–241.

[35] J. Chen, L. Wu, J. Zhang, L. Zhang, D. Gong, Y. Zhao, S. Hu, Y. Wang, X. Hu, B. Zheng, and K. Zhang, “Deep learning-based model for detecting 2019 novel coronavirus pneumonia on high-resolution computed tomography: A prospective study,” medRxiv, Jan. 2020. [Online]. Available: https://www.medrxiv.org/content/10.1101/2020.02.25.20021568v2 10.1038/s41598-020-76282-0 PMC7645624 33154542

[36] C. Zheng, X. Deng, Q. Fu, Q. Zhou, J. Feng, H. Ma, W. Liu, and X. Wang, “Deep learning-based detection for COVID-19 from chest CT using weak label,” medRxiv, Jan. 2020. [Online]. Available: https://www.medrxiv.org/content/10.1101/2020.03.12.20027185v2

[37] S. Jin, B. Wang, H. Xu, C. Luo, L. Wei, W. Zhao, X. Hou, W. Ma, Z. Xu, Z. Zheng, and W. Sun, “AI-assisted CT imaging analysis for COVID-19 screening: Building and deploying a medical ai system in four weeks,” medRxiv, Jan. 2020. [Online]. Available: https://www.medrxiv.org/content/10.1101/2020.03.19.20039354v1 10.1016/j.asoc.2020.106897 PMC7654325 33199977

[38] Z. Zhou, M. M. R. Siddiquee, N. Tajbakhsh, and J. Liang, “UNet++: A nested U-Net architecture for medical image segmentation,” in Deep Learning in Medical Image Analysis and Multimodal Learning for Clinical Decision Support. Cham, Switzerland: Springer, 2018, pp. 3–11. 10.1007/978-3-030-00889-5_1 PMC7329239 32613207

[39] F. Milletari, N. Navab, and S.-A. Ahmadi, “V-net: Fully convolutional neural networks for volumetric medical image segmentation,” in Proc. 4th Int. Conf. 3D Vis. (3DV), Oct. 2016, pp. 565–571.

[40] J. Long, E. Shelhamer, and T. Darrell, “Fully convolutional networks for semantic segmentation,” in Proc. IEEE Conf. Comput. Vis. Pattern Recognit. (CVPR), Jun. 2015, pp. 3431–3440. 10.1109/TPAMI.2016.2572683 27244717

[41] K. He, X. Zhang, S. Ren, and J. Sun, “Deep residual learning for image recognition,” in Proc. IEEE Conf. Comput. Vis. Pattern Recognit. (CVPR), Jun. 2016, pp. 770–778.

[42] Y. Chen, J. Li, H. Xiao, X. Jin, S. Yan, and J. Feng, “Dual path networks,” in Proc. Adv. Neural Inf. Process. Syst., 2017, pp. 4467–4475.

[43] C. Szegedy, V. Vanhoucke, S. Ioffe, J. Shlens, and Z. Wojna, “Rethinking the inception architecture for computer vision,” in Proc. IEEE Conf. Comput. Vis. Pattern Recognit. (CVPR), Jun. 2016, pp. 2818–2826.

[44] F. Wang, M. Jiang, C. Qian, S. Yang, C. Li, H. Zhang, X. Wang, and X. Tang, “Residual attention network for image classification,” in Proc. IEEE Conf. Comput. Vis. Pattern Recognit. (CVPR), Jul. 2017, pp. 3156–3164.

[45] C. Jin, W. Chen, Y. Cao, Z. Xu, X. Zhang, L. Deng, C. Zheng, J. Zhou, H. Shi, and J. Feng, “Development and evaluation of an AI system for COVID-19 diagnosis,” medRxiv, Jan. 2020. [Online]. Available: https://www.medrxiv.org/content/10.1101/2020.03.20.20039834v3 10.1038/s41467-020-18685-1 PMC7547659 33037212

[46] H. Shi, X. Han, N. Jiang, Y. Cao, O. Alwalid, J. Gu, Y. Fan, and C. Zheng, “Radiological findings from 81 patients with COVID-19 pneumonia in wuhan, China: A descriptive study,” Lancet Infectious Diseases, vol. 20, no. 4, pp. 425–434, Apr. 2020. 32105637 10.1016/S1473-3099(20)30086-4 PMC7159053

[47] W. Zhao, Z. Zhong, X. Xie, Q. Yu, and J. Liu, “Relation between chest CT findings and clinical conditions of coronavirus disease (COVID-19) pneumonia: A multicenter study,” Amer. J. Roentgenology, vol. 214, no. 5, pp. 1072–1077, 2020. 10.2214/AJR.20.22976 32125873

[48] S. Wang, B. Kang, J. Ma, X. Zeng, M. Xiao, J. Guo, M. Cai, J. Yang, Y. Li, X. Meng, and B. Xu, “A deep learning algorithm using CT images to screen for corona virus disease (COVID-19),” medRxiv, Jan. 2020. [Online]. Available: https://www.medrxiv.org/content/10.1101/2020.02.14.20023028v5 10.1007/s00330-021-07715-1 PMC7904034 33629156

[49] Y. Song, S. Zheng, L. Li, X. Zhang, X. Zhang, Z. Huang, J. Chen, H. Zhao, Y. Jie, R. Wang, and Y. Chong, “Deep learning enables accurate diagnosis of novel coronavirus (COVID-19) with CT images,” medRxiv, Jan. 2020. [Online]. Available: https://www.medrxiv.org/content/10.1101/2020.02.23.20026930v1 10.1109/TCBB.2021.3065361 PMC8851430 33705321

[50] F. Shi, L. Xia, F. Shan, D. Wu, Y. Wei, H. Yuan, H. Jiang, Y. Gao, H. Sui, and D. Shen, “Large-scale screening of COVID-19 from community acquired pneumonia using infection size-aware classification,” 2020, arXiv:2003.09860. [Online]. Available: http://arxiv.org/abs/2003.09860 10.1088/1361-6560/abe838 33729998

[51] X. Xu, X. Jiang, C. Ma, P. Du, X. Li, S. Lv, L. Yu, Y. Chen, J. Su, G. Lang, Y. Li, H. Zhao, K. Xu, L. Ruan, and W. Wu, “Deep learning system to screen coronavirus disease 2019 pneumonia,” 2020, arXiv:2002.09334. [Online]. Available: http://arxiv.org/abs/2002.09334 10.1016/j.eng.2020.04.010 PMC7320702 32837749

[52] F. Shan, Y. Gao, J. Wang, W. Shi, N. Shi, M. Han, Z. Xue, D. Shen, and Y. Shi, “Lung infection quantification of COVID-19 in CT images with deep learning,” 2020, arXiv:2003.04655. [Online]. Available: http://arxiv.org/abs/2003.04655

[53] Y. Xiong, D. Sun, Y. Liu, Y. Fan, L. Zhao, X. Li, and W. Zhu, “Clinical and high-resolution CT features of the COVID-19 infection: Comparison of the initial and follow-up changes,” Investigative Radiol., vol. 55, no. 6, pp. 332–339, Jun. 2020. 10.1097/RLI.0000000000000674 PMC7147282 32134800

[54] Z. Tang, W. Zhao, X. Xie, Z. Zhong, F. Shi, J. Liu, and D. Shen, “Severity assessment of coronavirus disease 2019 (COVID-19) using quantitative features from chest CT images,” 2020, arXiv:2003.11988. [Online]. Available: http://arxiv.org/abs/2003.11988

[55] S. Dodge and L. Karam, “Understanding how image quality affects deep neural networks,” in Proc. 8th Int. Conf. Qual. Multimedia Exper. (QoMEX), Jun. 2016, pp. 1–6.

[56] N. Satpute, R. Naseem, E. Pelanis, J. Gómez-Luna, F. A. Cheikh, O. J. Elle, and J. Olivares, “GPU acceleration of liver enhancement for tumor segmentation,” Comput. Methods Programs Biomed., vol. 184, Feb. 2020, Art. no. 105285. 10.1016/j.cmpb.2019.105285 31896055

[57] E. Reinhard, G. Ward, S. Pattanaik, and P. Debevec, High Dynamic Range Imaging: Acquisition, Display, and Image-Based Lighting (The Morgan Kaufmann Series in Computer Graphics). San Francisco, CA, USA: Morgan Kaufmann, 2005.

[58] D. Völgyes, A. Martinsen, A. Stray-Pedersen, D. Waaler, and M. Pedersen, “A weighted histogram-based tone mapping algorithm for CT images,” Algorithms, vol. 11, no. 8, p. 111, Jul. 2018.

[59] M. A. Campos and A. A. Diaz, “The role of computed tomography for the evaluation of lung disease in Alpha-1 antitrypsin deficiency,” Chest, vol. 153, no. 5, pp. 1240–1248, 5 2018. 29175361 10.1016/j.chest.2017.11.017 PMC6026284

[60] R. Janssen, Computational Image Quality, vol. PM101, R. Janssen, Ed. Bellingham, WA, USA: SPIE, 2001.

[61] S. Xie, R. Girshick, P. Dollar, Z. Tu, and K. He, “Aggregated residual transformations for deep neural networks,” in Proc. IEEE Conf. Comput. Vis. Pattern Recognit. (CVPR), Jul. 2017, pp. 1492–1500.

[62] S. Woo, J. Park, J.-Y. Lee, and I. S. Kweon, “CBAM: Convolutional block attention module,” in Proc. Eur. Conf. Comput. Vis., 2018, pp. 3–19.

[63] C. Szegedy, W. Liu, Y. Jia, P. Sermanet, S. Reed, D. Anguelov, D. Erhan, V. Vanhoucke, and A. Rabinovich, “Going deeper with convolutions,” in Proc. IEEE Conf. Comput. Vis. Pattern Recognit. (CVPR), Jun. 2015, pp. 1–9.

[64] D. Han, J. Kim, and J. Kim, “Deep pyramidal residual networks,” in Proc. IEEE Conf. Comput. Vis. Pattern Recognit. (CVPR), Jul. 2017, pp. 5927–5935.

[65] S. Zagoruyko and N. Komodakis, “Wide residual networks,” 2016, arXiv:1605.07146. [Online]. Available: http://arxiv.org/abs/1605.07146

[66] F. Chollet, “Xception: Deep learning with depthwise separable convolutions,” in Proc. IEEE Conf. Comput. Vis. Pattern Recognit. (CVPR), Jul. 2017, pp. 1251–1258.

[67] I. Goodfellow, Y. Bengio, A. Courville, and Y. Bengio, Deep Learning, vol. 1. Cambridge, MA, USA: MIT Press, 2016.

[68] S. Haykin, Neural Networks, vol. 2. New York, NY, USA: Prentice-Hall, 1994.

[69] K. Greff, R. K. Srivastava, J. Koutnik, B. R. Steunebrink, and J. Schmidhuber, “LSTM: A search space odyssey,” IEEE Trans. Neural Netw. Learn. Syst., vol. 28, no. 10, pp. 2222–2232, Oct. 2017. 27411231 10.1109/TNNLS.2016.2582924

[70] R. Jozefowicz, W. Zaremba, and I. Sutskever, “An empirical exploration of recurrent network architectures,” in Proc. Int. Conf. Mach. Learn., 2015, pp. 2342–2350.

[71] Colah. (2015). Understanding LSTM Networks. [Online]. Available: http://colah.github.io/posts/2015-08-Understanding-LSTMs/

[72] A. Karpathy, J. Johnson, and L. Fei-Fei, “Visualizing and understanding recurrent networks,” 2015, arXiv:1506.02078. [Online]. Available: http://arxiv.org/abs/1506.02078

[73] J. Paul Cohen, P. Morrison, and L. Dao, “COVID-19 image data collection,” 2020, arXiv:2003.11597. [Online]. Available: http://arxiv.org/abs/2003.11597

[74] F. Liao, M. Liang, Z. Li, X. Hu, and S. Song, “Evaluate the malignancy of pulmonary nodules using the 3-D deep leaky noisy-OR network,” IEEE Trans. Neural Netw. Learn. Syst., vol. 30, no. 11, pp. 3484–3495, Nov. 2019. 30794190 10.1109/TNNLS.2019.2892409

[75] B. Zhou, Q. Cui, X.-S. Wei, and Z.-M. Chen, “BBN: Bilateral-branch network with cumulative learning for long-tailed visual recognition,” 2019, arXiv:1912.02413. [Online]. Available: http://arxiv.org/abs/1912.02413

[76] M. Buda, A. Maki, and M. A. Mazurowski, “A systematic study of the class imbalance problem in convolutional neural networks,” Neural Netw., vol. 106, pp. 249–259, Oct. 2018. 30092410 10.1016/j.neunet.2018.07.011

[77] A. Paszke, S. Gross, F. Massa, A. Lerer, J. Bradbury, G. Chanan, T. Killeen, Z. Lin, N. Gimelshein, L. Antiga, and A. Desmaison, “Pytorch: An imperative style, high-performance deep learning library,” in Proc. Adv. Neural Inf. Process. Syst., 2019, pp. 8024–8035.

[78] D. P. Kingma and J. Ba, “Adam: A method for stochastic optimization,” 2014, arXiv:1412.6980. [Online]. Available: http://arxiv.org/abs/1412.6980

[79] L. N. Smith, “Cyclical learning rates for training neural networks,” in Proc. IEEE Winter Conf. Appl. Comput. Vis. (WACV), Mar. 2017, pp. 464–472.

